# Acid-base synergistic activation of coal gasification fine slag into hierarchical porous carbon for enhanced Cr(vi) adsorption and reduction

**DOI:** 10.1039/d6ra02711c

**Published:** 2026-05-18

**Authors:** Lijuan Bai, Hua Wang, Kaipeng Guo, Xia Li, Kaiwen Bai, Zhengyan Shi, Rui Dang

**Affiliations:** a College of Chemistry and Chemical Engineering, Yulin University Yulin City 719000 China 99452715@qq.com; b Shaanxi Provincial Key Laboratory of Clean Utilization of Low-Modified Coal, Yulin University Yulin City 719000 China

## Abstract

A sequential acid–alkali activation strategy was developed to convert coal gasification fine slag (CGFS) into a hierarchical porous carbon (FC) for efficient Cr(vi) removal. During the process, mineral ash was removed by acid leaching to expose encapsulated carbon domains, while subsequent alkali activation disrupted silicate frameworks and reconstructed interconnected micro–meso–macroporous networks, producing a porous carbon with a high specific surface area of 630.3 m^2^ g^−1^. Benefiting from the optimized pore architecture and heteroatom-enriched surface chemistry, FC exhibited excellent affinity toward Cr(vi), achieving a maximum adsorption capacity of 179.9 mg g^−1^ at 318 K and pH 2. Adsorption kinetics and isotherm analyses indicated monolayer adsorption behavior, with chemisorption serving as the rate-limiting step, while thermodynamic parameters suggested that the process was spontaneous and endothermic. Coexisting anions imposed varying degrees of competitive inhibition on Cr(vi) adsorption in the order of SO_4_^2−^ > HPO_4_^2−^ > CO_3_^2−^ > NO_3_^−^ > Cl^−^; nevertheless, considerable removal efficiency was maintained in multi-ion systems, and 63.32% of the initial performance was retained after five regeneration cycles. Mechanistic analyses revealed that Cr(vi) removal was governed by synergistic processes involving pore-filling enrichment, electrostatic attraction and hydrogen bonding, as well as surface redox reactions and complexation. These findings demonstrate the feasibility of valorizing CGFS as a low-cost precursor for advanced adsorbents in heavy-metal wastewater remediation.

## Abstract 2

Hierarchical porous carbon from coal gasification fine slag *via* acid–alkali activation exhibited a high surface area (630.3 m^2^ g^−1^) and Cr(vi) adsorption capacity of 179.9 mg g^−1^ through pore filling, electrostatic attraction and redox reactions.

## Graphical abstract

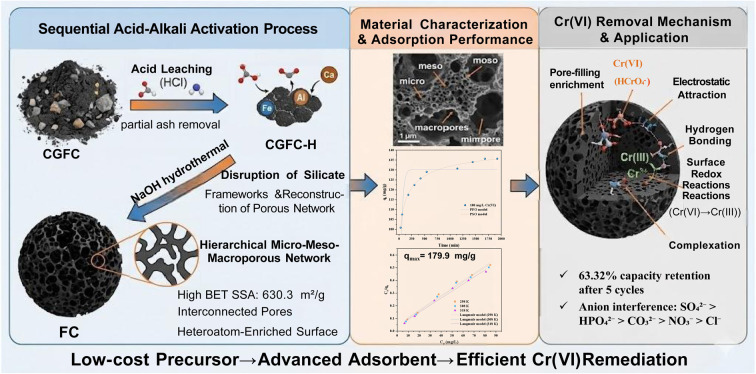

## 1 Introduction

The accelerating pace of industrialization has led to the widespread release of heavy metal ions into aquatic environments, primarily from mining operations, electroplating, leather tanning, and metallurgical processes.^1^ Heavy metals are non-biodegradable, tend to persist in ecosystems, and accumulate through the food chain, posing chronic risks to reproductive and immune functions in both wildlife and humans.^2^ Among these contaminants, chromium (Cr) is of particular concern. It predominantly occurs in two stable oxidation states: Cr(vi) and Cr(iii). Extensive research has demonstrated that Cr(vi) exhibits toxicity approximately three orders of magnitude greater than Cr(iii), with well-documented carcinogenic and mutagenic properties.^3^ Given the stringent regulatory limits on Cr(vi) discharge and its widespread occurrence in industrial effluents, the development of effective and scalable strategies for Cr(vi) removal remains an urgent environmental priority.

Among the various remediation technologies available-including chemical precipitation, ion exchange, membrane filtration, and photocatalytic reduction-adsorption has emerged as a particularly attractive approach owing to its operational simplicity, low energy consumption, and adaptability to diverse wastewater compositions.^4^ The performance of adsorption-based systems is fundamentally governed by the physicochemical properties of the adsorbent, driving sustained interest in low-cost, high-performance materials derived from industrial solid wastes. CGFS, a predominant solid byproduct of coal chemical industries in China, is generated at an annual rate exceeding 70 million tonnes^5^ and is predominantly disposed of by stockpiling or landfilling, causing resource inefficiency and environmental burden. Comparable residues accumulate in coal gasification operations worldwide. CGFS is compositionally rich in quartz, amorphous aluminosilicates, and residual carbon,^6^ and its inherent porous architecture and reactive carbonaceous framework position it as a promising precursor for engineered adsorbents. Converting CGFS into functional porous carbon materials therefore simultaneously addresses two pressing challenges: the valorization of a problematic industrial waste stream and the provision of cost-effective adsorbents for heavy-metal wastewater treatment.

Porous carbons are commonly synthesized *via* hard-template methods, chemical activation, and physical activation. Hard-template approaches can precisely regulate pore structure, but they usually rely on sacrificial and expensive templates and involve complicated post-removal procedures.^7^ Chemical activation using agents such as KOH, NaOH, or ZnCl_2_ is effective for generating abundant micropores, yet the strong corrosivity of activators and secondary pollution remain major concerns.^8^ Physical activation (*e.g.*, steam or CO_2_ activation) is relatively cleaner, but it generally requires high temperatures and often suffers from low carbon yield and insufficient pore development.^9^ Recent studies have shown that sequential acid leaching and alkali activation can significantly improve pore accessibility and adsorption performance of coal-derived solid wastes. For example, acid-assisted demineralization effectively removes ash and exposes blocked carbon surfaces, while subsequent alkaline treatment promotes hierarchical pore formation and enhances active-site exposure.^10–12^ Notably, CGFS-derived porous carbons have exhibited remarkable potential in Cr(vi) sequestration. Investigations reveal that acid leaching markedly augments adsorption capacity by removing soluble ash, whereas hydrothermal or oxidative treatments foster interconnected pore networks.^10,11^ Furthermore, porous carbons synthesized *via* high-temperature alkali activation after flotation-based carbon extraction display enhanced porosity.^13^ However, several critical bottlenecks hinder the large-scale application of these methodologies. Traditional single-step activation often fails to fully decouple the complex carbon-encapsulated mineral matrices inherent in CGFS, resulting in “dead pores” and restricted mass transfer.^14^ Moreover, the atom-level synergy between pore structure and surface chemistry remains poorly understood; specifically, how heteroatoms (*e.g.*, O and S) within the CGFS carbon skeleton mediate the electron transfer required for Cr(vi) reduction to the less toxic Cr(iii).^15^ While the influence of coexisting ions in complex aquatic matrices often suppresses reduction kinetics, few studies have addressed how to maintain surface reactivity under such competitive conditions.^16^ Additionally, the energy-intensive nature of high-temperature alkali activation diminishes its economic viability. Therefore, there exists a pressing imperative to devise a low-energy, synergistic activation protocol that optimizes both pore interconnectivity and surface redox activity.

To surmount these limitations, this study introduces an integrated acid-base synergistic demineralization and pore-engineering strategy. Unlike conventional methods, our approach employs a sequential hydrochloric acid-sodium hydroxide tandem process. The initial acid leaching selectively dissolves occlusive metallic oxides (*e.g.*, Fe_2_O_3_, Al_2_O_3_), effectively “unzipping” the carbon-mineral boundaries. This is followed by a mild NaOH activation step that etches the silicate residues and disrupts Si–O/Si–Si bonds, simultaneously grafting oxygen-containing functional groups and creating a highly accessible hierarchical micro-*meso*-macroporous configuration. This tandem methodology not only unveils previously inaccessible reactive sites but also preserves the intrinsic heteroatoms (O/S) for enhanced redox-assisted adsorption. The efficacy of this strategy was systematically evaluated across four dimensions: (1) the structural evolution from raw slag to hierarchical carbon (FC) through demineralization; (2) Cr(vi) adsorption-reduction dynamics, encompassing kinetics, isotherms, and thermodynamics; (3) selectivity and durability in multi-anion environments; and (4) the molecular-level surface reaction mechanisms driving the adsorption-reduction synergy. These insights provide a scalable and sustainable paradigm for the high-value valorization of CGFS in industrial wastewater remediation.

## 2 Materials and methods

### 2.1 Materials and reagents

The CGFS samples were provided by Shaanxi Coal Chemical Industry Group Co., Ltd. The samples originated from a typical industrial entrained-flow coal gasifier operating at high temperature (generally above 1200 °C), using bituminous coal from the local coalfield as feedstock-operating conditions that are representative of the dominant coal-gasification technology deployed in northwestern China. To ensure consistency in subsequent characterization and experiments, the raw CGFS was first dried at 105 °C until a constant weight was achieved. As shown in Table 1, proximate analysis (GB/T 212–2008) indicated that the dried CGFS contained 21.72 wt% residual carbon. The relatively high residual carbon content demonstrates that CGFS possesses abundant carbonaceous components, making it a promising precursor for the preparation of porous carbon-based adsorbents. In addition, the mineral-rich ash fraction is expected to facilitate pore structure development during the subsequent acid leaching and activation processes, thereby enhancing the adsorption performance of the final material.

**Table 1:** Proximate analysis of dried CGFS

Sample	Ash content (wt%)	Volatile matter (wt%)	Residual carbon content (wt%)
Dried CGFS	77.27	1.01	21.72

The chemical reagents used in the experiment include hydrochloric acid (HCl), sodium hydroxide (NaOH), potassium dichromate (K_2_Cr_2_O_7_), sodium chloride (NaCl), sodium sulfate (Na_2_SO_4_), sodium carbonate (Na_2_CO_3_), sodium nitrate (NaNO_3_), and sodium hydrogen phosphate (Na_2_HPO_4_), all of which are of analytical grade. Purchased from Tianjin Comiao Chemical Reagent Co., LTD.

### 2.2 Material preparation

The preparation procedure is illustrated in Fig. S1.

Pretreatment: ten grams of 200-mesh CGFS were ultrasonically cleaned with deionized water at least three times, with each ultrasonic cycle lasting 15 min, followed by suction filtration.

Acid treatment: the pretreated sample (10 g) was stirred with 400 mL of 10% HCl at 343 K for 2 h, after which solid–liquid separation was performed *via* suction filtration. The filter cake was repeatedly washed with deionized water until the filtrate reached neutral pH and subsequently dried at 378 K overnight to obtain the acid-treated residue, denoted as CGFS-H.

Alkali treatment: the acid-treated residue was mixed with 400 mL of 2 mol L^−1^ NaOH solution in a polytetrafluoroethylene (PTFE) beaker and stirred at 363 K for 6 h. The resulting solid was collected by filtration, washed with deionized water until neutral, and dried at 378 K for 12 h. The final product was labeled as FC.

### 2.3 Characterization methods

Field emission scanning electron microscopy (SEM, SIGMA 300) was used to observe the surface morphology of the samples at different magnifications to obtain detailed microstructure characteristics. Meanwhile, the energy dispersive spectrometer (EDS) equipped on the scanning electron microscope is a device used to analyze the elemental composition and distribution on the sample surface. The mineral phase composition of the sample was analyzed by X-ray diffraction (XRD, Bruker D8 Advance) using a Cu-Kα radiation source with a scanning range of 10° to 80° and a step size of 0.02°. The relevant calculation and analysis were completed using Jade 6 software. The composition of surface functional groups was analyzed by Fourier Transform Infrared Spectroscopy (FTIR, Bruker Tensor 27), and detected by the KBr pellet method. The wavenumber range was 4000 cm^−1^ to 400 cm^−1^, and the resolution was 4 cm^−1^. The surface chemical composition of the samples was detected by X-ray photoelectron spectroscopy (XPS, Thermo Scientific K-Alpha). The analysis chamber pressure was lower than 2.0 × 10^−7^ mbar, and a monochromatic Al Kα X-ray source was used. The full-spectrum scanning flux energy is 150 eV and the step size is 1 eV. The narrow-band scanning flux energy is 50 eV and the step size is 0.1 eV. The nitrogen adsorption–desorption isotherms were measured using a nitrogen adsorption analyzer (ASAP 2460), and the specific surface area and pore structure of the samples were determined by BET (Brunauer–Emmett–Teller) and BJH (Barrett–Joyner–Halenda) methods. The micropore surface area was quantified by the *t*-plot method, while the mesoporous surface area was determined by the BJH method. Raman spectroscopy (Horiba Lab RAM HR Evolution) is used to analyze the microstructure of carbon in materials. The laser excitation wavelength is 532 nm, and the spectral resolution is better than 1 cm^−1^.

### 2.4 Adsorption experiment

#### 2.4.1 Cr(vi) adsorption experiment

To explore the influencing factors of the adsorption effect of Cr(vi), this study systematically investigated the initial concentration (50–150 mg L^−1^), adsorption time (30–1920 min), temperature (298–318 K), pH (1–10), adsorbent dosage (0.02–0.10 g), and regeneration times (1–5 times). The concentration was determined at a wavelength of 540 nm by the diphenylcarbazide spectrophotometry. All experiments were conducted three times in parallel to control the error.

The adsorption capacity (*q*_*t*_) and Cr(vi) removal efficiency (*R*) at the given time were determined respectively using eqn (1) and (2).1
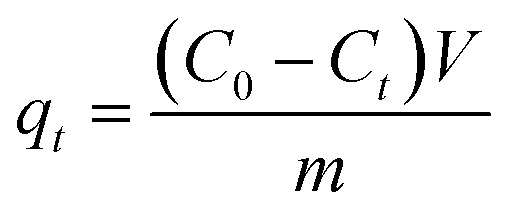
2
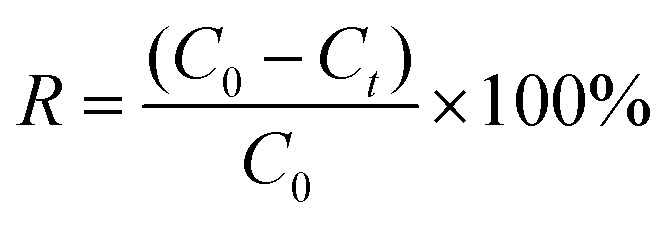


Among them, *q*_*t*_ (mg g^−1^) represents the adsorption capacity of the adsorbent at time *t*; C_0_ (mg L^−1^) and *C*_*t*_ (mg L^−1^) represent the initial concentration of Cr(vi) and the concentration at time *t*, respectively. The removal efficiency of Cr(vi) by the adsorbent is expressed as *R* (%). When adsorption reaches equilibrium, the equilibrium concentration of Cr(vi) in the solution is *C*_e_ (mg L^−1^), and the corresponding adsorption capacity is *q*_e_ (mg g^−1^).

#### 2.4.2 Repeatability experiments of FC

Using a 0.05 mol L^−1^ sodium hydroxide solution as the eluent, the saturated adsorbed FC was treated in an ultrasonic cleaner at a solid–liquid ratio of 5 : 2 (m V^−1^, mg mL^−1^) for 15 minutes. After desorption was repeated three times, the regeneration efficiency of the material was determined.

#### 2.4.3 Experiment on the adsorption of Cr(vi) by coexisting anions on FC

To explore the removal efficiency (*C*_*t*_/*C*_0_) of Cr(vi) in solutions containing five common anions (Cl^−^, SO_4_^2−^, NO_3_^−^, HPO_4_^2−^ and CO_3_^2−^, each with a concentration of 0.01 M) under 308 K conditions, where *C*_*t*_ represents the concentration of Cr(vi) at a specific moment and *C*_0_ is the initial concentration. The volume of Cr(vi) solution is 50 mL (50 mg L^−1^), and the pH value of the solution is not adjusted. The dosage of adsorbent is 0.02 g.

## 3 Results and discussion

### 3.1 Structural characterization of materials

#### 3.1.1 Microscopic morphology analysis

**Fig. 1:**
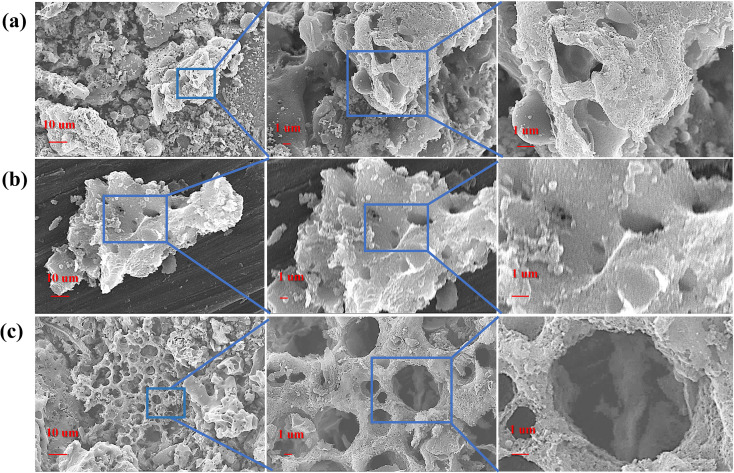
Surface morphology of (a) CGFS, (b) CGFS-H and (c) FC.

Fig. 1a presents the surface morphology of the raw CGFS. The material is primarily composed of spherical inorganic mineral particles and residual carbon. Local magnification reveals diverse morphological characteristics between these components. Most inorganic mineral particles exhibit smooth surfaces without an observable pore structure. Numerous submicron spherical particles are aggregated on the surface of the residual carbon or adhered to large inorganic particles, whereas the larger particles are predominantly present in a dispersed state.^17^ In contrast, the residual carbon displays a well-developed porous structure characterized by abundant micrometer-scale and submicrometer-scale pores and a relatively rough surface. Additionally, some inorganic particles are embedded within the carbon pores, obstructing pore channels and consequently reducing pore size. Fig. 1b presents the SEM image of CGFS-H. After acid treatment, the spherical inorganic mineral particles almost completely disappear, while the surface still exhibits a relatively rough morphology. Fig. 1c presents the microstructure of FC after the combined acid–alkali treatment. The inorganic particles previously attached to the carbon surface or embedded within the pores are nearly completely removed, indicating the effective dissolution of mineral phases. Meanwhile, the carbon framework remains largely preserved, accompanied by an increase in pore size and the predominant formation of more open pore channels. Moreover, abundant newly generated submicrometer-scale pores are observed on the surface and pore walls. Consequently, a hierarchical porous architecture with interconnected pore channels is developed, which can significantly facilitate mass transfer and improve the accessibility of adsorption sites.

#### 3.1.2 Phase composition analysis

**Fig. 2:**
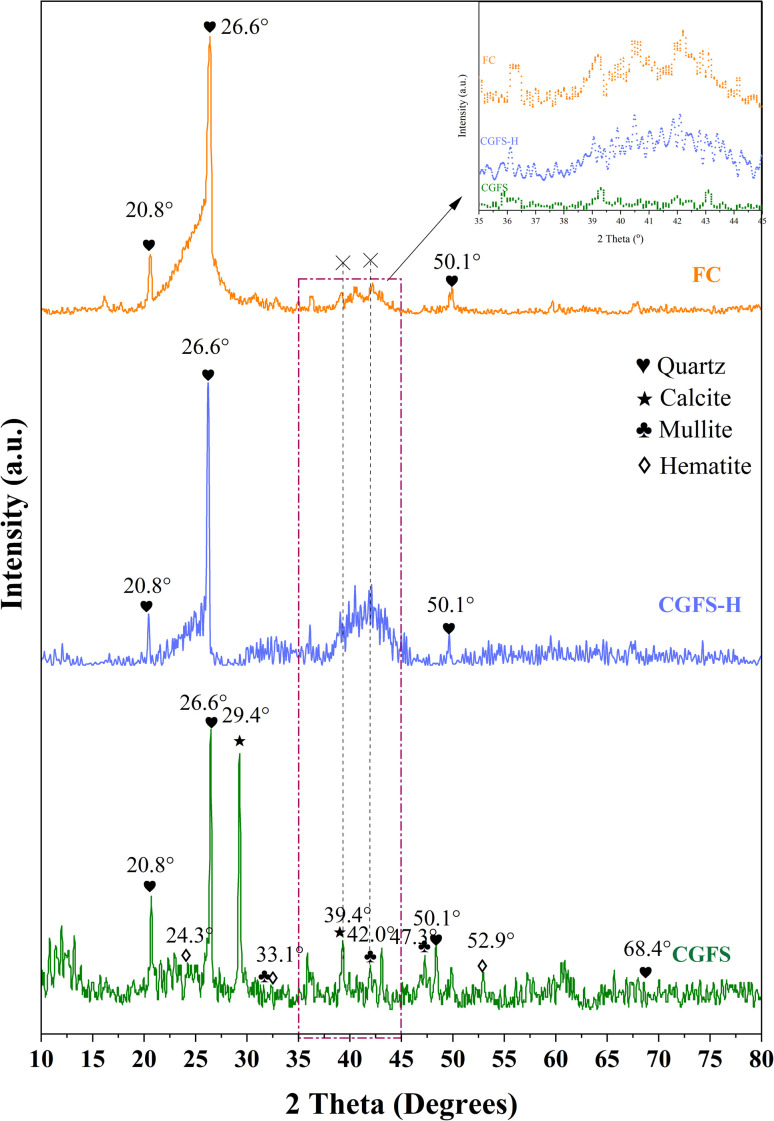
XRD patterns of CGFS, CGFS-H and FC.

Fig. 2 compares the XRD patterns of CGFS, CGFS-H, and FC. The raw CGFS exhibits several sharp diffraction peaks at 20.8°, 26.5°, 48.4°, and 68.4°, corresponding to crystalline quartz (SiO_2_).^18^ In addition, diffraction peaks attributed to Al_2_O_3_, CaO, and Fe_2_O_3_ are also observed. After acid treatment, pronounced changes occur in the XRD pattern of CGFS-H, and similar features are retained in FC following the subsequent NaOH treatment. Specifically, a broadened diffraction peak appears at approximately 42.2° (indicated by the red dashed box), accompanied by a characteristic “steamed-bun-shaped” hump in the 2*θ* range of 20°–30°,^19^ indicating enrichment and exposure of the intrinsic amorphous carbon phase in both CGFS-H and FC.^20^ Meanwhile, the diffraction peak of SiO_2_ at 26.5° is still detectable, suggesting that a small fraction of quartz remains after the acid–alkali treatment. The residual SiO_2_ can interact with the carbon framework, inducing local structural heterogeneity and consequently affecting the evolution of surface morphology and pore structure, which is consistent with the SEM observations.

#### 3.1.3 Functional group analysis

**Fig. 3:**
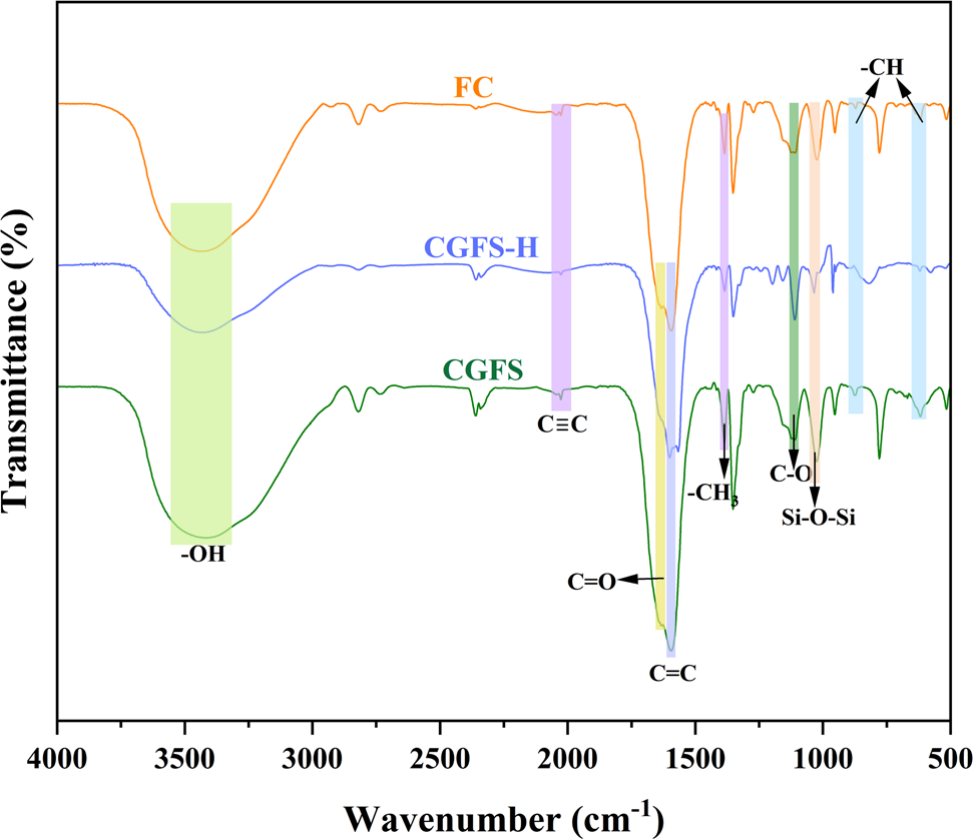
FTIR spectra of CGFS, CGFS-H and FC.

Fig. 3 presents the FTIR spectra of FC, CGFS-H, and CGFS, clearly revealing the evolution of surface functional groups during the modification process. All samples exhibit a broad and intense absorption band around 3440 cm^−1^, which is attributed to the stretching vibration of hydroxyl groups (–OH).^21^ Notably, the intensity of this band in CGFS is significantly stronger than that in CGFS-H and FC, suggesting a higher density of oxygen-containing functional groups (*e.g.*, hydroxyl and carboxyl groups) on the surface of the raw material. These groups can serve as important active sites for ion adsorption. A distinct absorption peak observed at approximately 2030 cm^−1^ corresponds to the stretching vibration of C≡C bonds.^22^ The absorption band located in the range of 1700–1600 cm^−1^ is assigned to the C=O stretching vibration of carboxyl groups. Combined with the broad –OH band mentioned above, this feature indicates the presence of oxygen-containing functionalities on the carbon framework. From CGFS to CGFS-H and FC, the pronounced attenuation of these peaks suggests that the acid–alkali treatment induces redistribution or partial consumption of oxygen-containing species, thereby modifying the surface chemical environment.^23^ In addition, the skeletal structure of the materials is characterized by the aromatic C=C stretching vibrations in the range of 1590–1500 cm^−1^ and the out-of-plane bending vibrations of aromatic C–H at 878 and 617 cm^−1^.^24^ The persistence of these peaks after modification indicates that the aromatic carbon framework remains largely stable under the treatment conditions and retains a conjugated π-electron structure, which is favorable for interfacial electron transfer.^25^ In the fingerprint region, the absorption peaks at 1388 cm^−1^ (–CH_3_) and 1112 cm^−1^ (C–O) remain essentially unchanged.^26^ Finally, the band at 1027 cm^−1^ is attributed to the stretching vibrations of Si–O–Si and Si–O bonds,^27^ suggesting that a portion of the siliceous components is retained after the acid–alkali treatment.

#### 3.1.4 Pore structure analysis

**Fig. 4:**
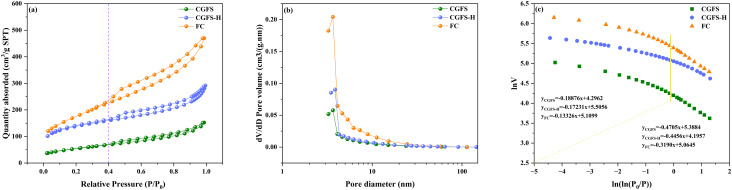
(a) N_2_ adsorption–desorption isotherms for CGFS, CGFS-H, and FC; (b) pore size distribution; (c) fractal dimension calculation.

Fig. 4a illustrates the N_2_ adsorption–desorption isotherms of CGFS, FC, and CGFS-H. In the low relative pressure range (*P*/*P*_0_ = 0–0.05), the adsorption capacities of FC and CGFS-H increase sharply with increasing pressure, exhibiting a pronounced micropore filling effect and confirming the abundance of microporous structures within the materials. When *P*/*P*_0_ > 0.4, all three samples display typical IUPAC type IV isotherm characteristics, accompanied by an H3-type hysteresis loop resulting from capillary condensation in mesopores, indicating the presence of slit-shaped mesoporous structures within the materials.^28^ A comparison reveals that the hysteresis loop of FC is significantly wider than those of CGFS-H and CGFS. Moreover, the isotherm of FC continues to rise when *P*/*P*_0_ > 0.9, suggesting the formation of a hierarchical pore architecture characterized by the cooperative development of micropores, mesopores, and macropores. Notably, the isotherm of FC consistently remains above those of the other samples, further demonstrating the superiority of the synergistic treatment strategy in constructing an efficient pore structure.

Analysis of the pore size distribution in Fig. 4b reveals that FC and CGFS-H exhibit a hierarchical pore structure comprising micropores, mesopores, and macropores, with the majority of pores concentrated in the mesoporous region. Furthermore, FC possesses the highest number of mesopores. Table 2 indicates that the specific surface areas of CGFS, CGFS-H, and FC are 191.2 m^2^ g^−1^, 593.0 m^2^ g^−1^, and 630.3 m^2^ g^−1^ respectively. FC exhibits the highest specific surface area and pore volume, which can be attributed to the synergistic acid–alkali treatment. This process effectively removes a large fraction of inorganic ash that blocks the residual carbon pores, thereby interconnecting previously isolated pores and exposing the intrinsic pore structure. In addition, the partial dissolution of silica species, compared with CGFS-H, further promotes pore development and enriches the hierarchical pore architecture.

**Table 2:** Specific surface area and pore distribution of CGFS, FC and CGFS-H

Samples	Specific surface area (m^2^ g^−1^)	Mesopore surface area (m^2^ g^−1^)	Micropore surface area (m^2^ g^−1^)	Total pore volume (cm^3^ g^−1^)	Aperture diameter (nm)
CGFS	191.272	75.175	28.082	0.236	4.925
CGFS-H	592.993	258.356	20.349	0.481	4.841
FC	630.308	250.078	63.794	0.728	4.623

The Frenkel–Halsey–Hill (FHH) (Text S1) model was applied to linearly fit the N_2_ adsorption data (Fig. 4c). A clear inflection point was observed in the relationship between ln(*V*) − ln (ln (*P*/*P*_0_)) at *P*/*P*_0_ = 0.4, indicating the fractal characteristics of the material surface.^29^ Using this inflection point as the boundary, the calculated fractal dimensions *D*_1_ (low-pressure region) and *D*_2_ (high-pressure region) represent the surface roughness of micropores and the structural complexity of meso/macropores, respectively (Table S1). The results show that both *D*_1_ (2.8668) and *D*_2_ (2.6810) of FC are higher than those of CGFS-H and CGFS, which is highly consistent with the observed variations in specific surface area and pore volume. The increase in fractal dimension quantitatively demonstrates that the synergistic treatment not only promotes pore development but also enhances the geometric complexity of the pore-wall surfaces, thereby providing FC with more abundant active sites and a more developed hierarchical pore structure.

#### 3.1.5 Analysis of carbon microcrystalline structure

**Fig. 5:**
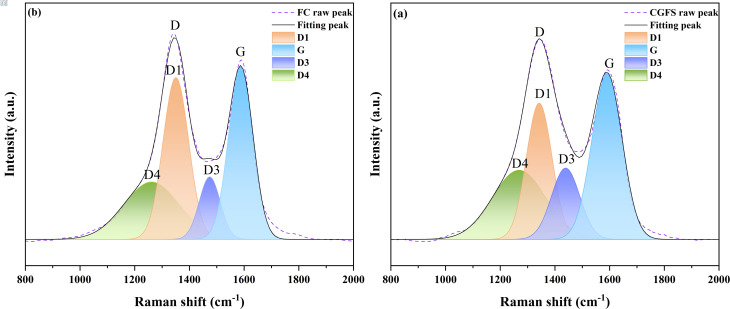
Fitted Raman spectra of (a) CGFS and (b) FC.

Fig. 5a and b present the Raman spectra of CGFS and FC. The two characteristic peaks at 1350 cm^−1^ and 1580 cm^−1^ correspond to the D and G bands respectively. The D band is characteristic of disordered graphite, whilst the G band originates from vibrations of the ideal graphite lattice. The peak intensity ratio *I*_D_/*I*_G_ reflects the degree of disorder or graphitisation within the carbon microcrystalline structure.^30^ The Raman spectra of each sample underwent deconvolution processing and were fitted to four peaks: three Lorentzian peaks (*D*_1_ (1350 cm^−1^), *D*_4_ (1200 cm^−1^), G (1580 cm^−1^)) and one Gaussian peak (*D*_3_ (1500 cm^−1^)).^31^ The *D*_1_ band correlates with in-plane defects induced by heteroatoms, reflecting vibrational modes of the graphite lattice; the *D*_3_ band originates from sp^2^ bonds in amorphous carbon, encompassing structurally irregular organic molecules and functional groups;^32^ while the *D*_4_ band corresponds to sp^2^–sp^3^ bonds at lattice edges or C–C and C=C stretching vibrations, forming a polyolefin-like structure.^33^ According to the quantitative parameters in Table S2, the ratios *A*_D1_/*A*_all_ and *A*_(*D*3+*D*4)_/*A*_all_ serve as indicators of structural disorder and active site density, while *A*_G_/*A*_all_ reflects the degree of lattice ordering.^32,33^ The results show that FC exhibits a higher *I*_D_/*I*_G_ ratio and a decreased *A*_G_/*A*_all_ compared to CGFS, indicating a reduction in graphitization and an increase in structural defects. This transformation is primarily driven by the NaOH etching process, which simultaneously promotes micropore formation and disrupts the long-range periodicity of the carbon layers, thereby inducing sp^2^–sp^3^ defects and amorphous carbon species that enhance the overall structural complexity of the FC material.

#### 3.1.6 Mechanism of ash removal

**Fig. 6:**
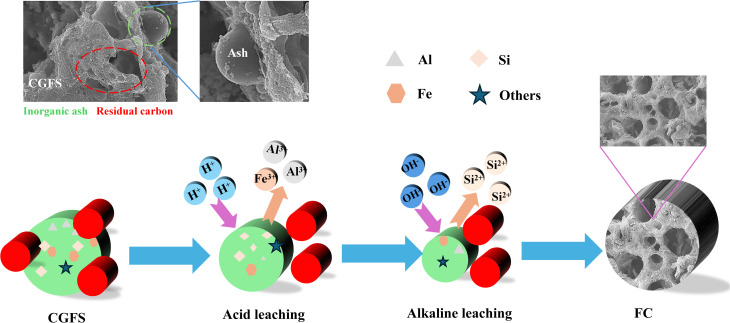
Schematic diagram of FC gray carbon extraction mechanism.

Fig. 6 illustrates the mechanism of ash separation in CGFS through combined acid-alkali treatment. As shown, during acid leaching, HCl dissolves soluble substances within the CGFS, causing internal ash particles to detach and dissolve from the carbon layer surface. Subsequently, during the alkaline leaching stage, NaOH undergoes neutralisation reactions with Si–OH groups on the CGFS surface while disrupting Si–Si bonds. This facilitates the formation of novel structures, thereby achieving pore creation.^34^ Concurrently, the sample undergoes vacuum filtration, washing, and drying to yield FC porous carbon material with a high specific surface area. Potential chemical reactions occurring during the combined acid-alkali process are outlined below (Eqn (3)–(7)).

Acid leaching process:

3 6HCl + Al_2_O_3_ → 3H_2_O + 2AlCl_3_

4 6HCl + Fe_2_O_3_ → 3H_2_O + 2FeCl_3_

5 2HCl + CaCO_3_ → H_2_O + CaCl_2_ + CO_2_↑

Alkali leaching process:

6 2NaOH + SiO_2_ → Na_2_SiO_3_ + H_2_O

7 2NaOH + Al_2_O_3_ → 2NaAlO_3_ + H_2_O

### 3.2 Evaluation of the adsorption performance of FC for Cr(vi)

#### 3.2.1 Adsorption characteristics of FC

**Fig. 7:**
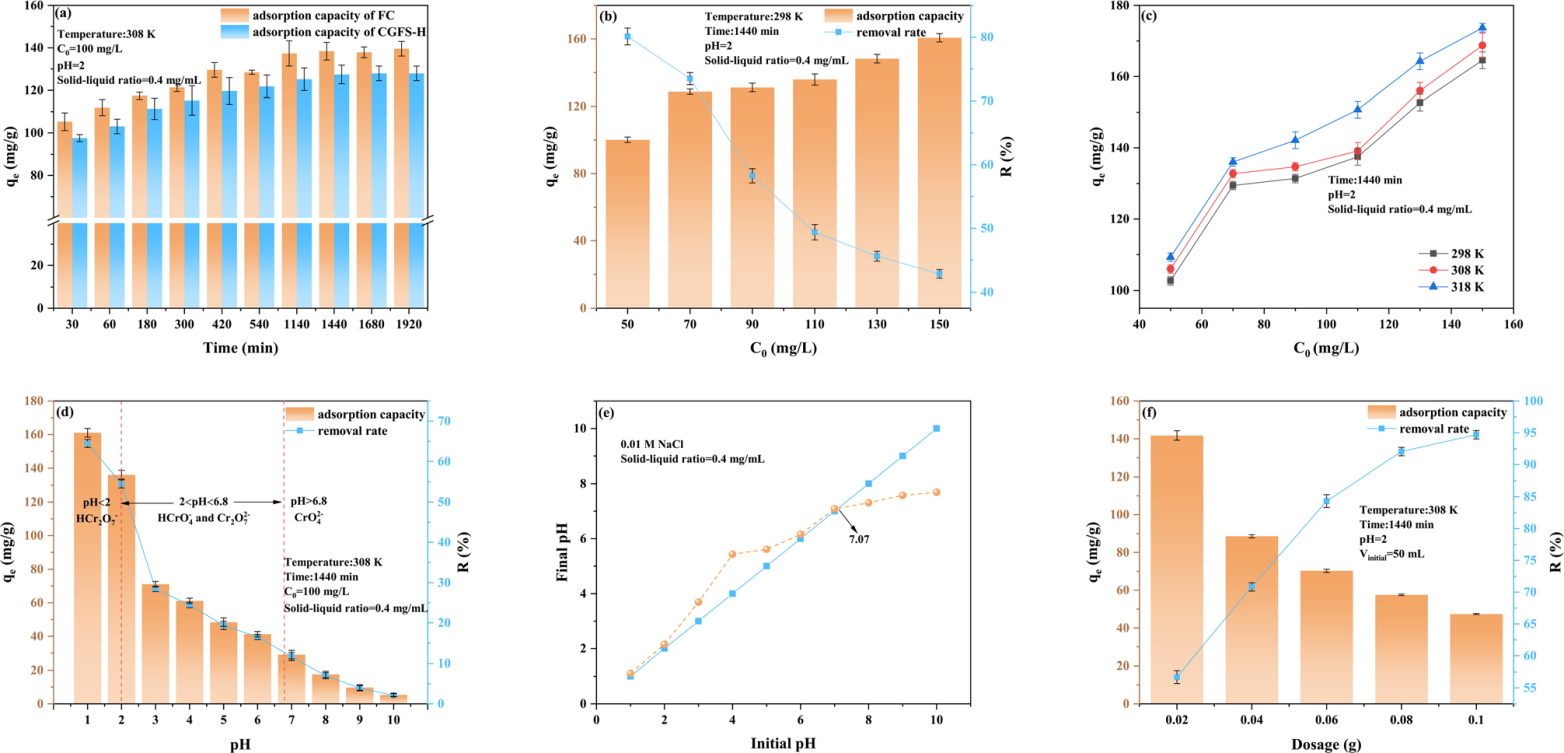
(a) Effect of contact time; (b) effect of initial concentration; (c) effect of initial concentration at different temperatures; (d) effect of pH value; (e) point of zero charge; (f) effect of dosage.

Under identical experimental conditions, FC exhibited a consistently higher adsorption capacity for Cr(vi) than CGFS-H throughout the adsorption process (Fig. 7a), indicating that FC possesses superior adsorption performance. During the initial adsorption stage (0–420 min), the FC surface contained a large number of unoccupied active sites, while the relatively high Cr(vi) concentration in the solution generated a substantial concentration gradient. This gradient significantly facilitated the mass transfer of Cr(vi) from the solution to the adsorbent surface, resulting in a rapid adsorption rate.^35^ As the adsorption process progressed, the active sites on the FC surface were gradually occupied by Cr(vi), and the Cr(vi) concentration in the solution simultaneously decreased. Consequently, the mass transfer driving force weakened, leading to a gradual decline in the adsorption rate until adsorption equilibrium was ultimately reached. Further examination of the effect of initial concentration (Fig. 7b) revealed that when the Cr(vi) concentration increased from 50 mg L^−1^ to 150 mg L^−1^, the adsorption capacity of FC increased significantly from approximately 100 mg g^−1^ to approximately 161 mg g^−1^. This enhancement can primarily be attributed to the increased concentration gradient, which strengthens the mass transfer and interaction between Cr(vi) species and the adsorbent surface.^36^ However, the removal efficiency decreased with increasing initial concentration, which is likely due to the limited number of available active sites on the FC surface that progressively approach saturation under higher concentration conditions. In addition, within the temperature range of 298–318 K, the adsorption capacity of FC toward Cr(vi) increased with increasing temperature (Fig. 7c), indicating that elevated temperature is favorable for the adsorption process.

Solution pH plays a critical role in influencing both the speciation of Cr(vi) and the surface charge characteristics of the adsorbent.^37^ As illustrated in Fig. 7d, when the pH increased from 1 to 10, the removal efficiency of Cr(vi) sharply decreased from 64% to 2%. Under strongly acidic conditions (pH < 2), Cr(vi) predominantly exists in the form of HCr_2_O_7_^−^. Within the pH range of 2–6.8, the dominant species are HCrO_4_^−^ and Cr_2_O_7_^2−^, whereas at pH values greater than 6.8, Cr(vi) mainly occurs as CrO_4_^2−^.^38^ The point of zero charge (pH_pzc_) of FC was determined to be 7.07 (Fig. 7e). When the solution pH is lower than this value, the –COOH and –OH functional groups on the FC surface undergo protonation to form –COOH_2_^+^ and –OH_2_^+^, resulting in a positively charged surface that promotes the adsorption of anionic Cr(vi) species through electrostatic attraction.^39^ Conversely, when the solution pH exceeds 7.07, the surface functional groups become deprotonated, rendering the FC surface negatively charged. Under these conditions, electrostatic repulsion occurs between the negatively charged surface and Cr(vi) anions. In addition, –OH present in the solution compete with CrO_4_^2−^ and other anionic species for adsorption sites, thereby significantly inhibiting Cr(vi) adsorption. Furthermore, the dosage of the adsorbent exerts a pronounced influence on the removal efficiency of Cr(vi) (Fig. 7f). When the FC dosage was 0.02 g, the removal efficiency was only approximately 57%, primarily due to the limited number of available adsorption sites on the adsorbent surface. As the dosage increased to 0.10 g, the removal efficiency significantly improved to approximately 95%, which can be attributed to the increased effective contact area and the greater number of available adsorption sites within the system. However, with further increases in adsorbent dosage, the adsorption capacity per unit mass exhibited a decreasing trend, which may be associated with particle aggregation that reduces the effective specific surface area, as well as the incomplete utilization of some active sites.

#### 3.2.2 Ionic interference and reusability

**Fig. 8:**
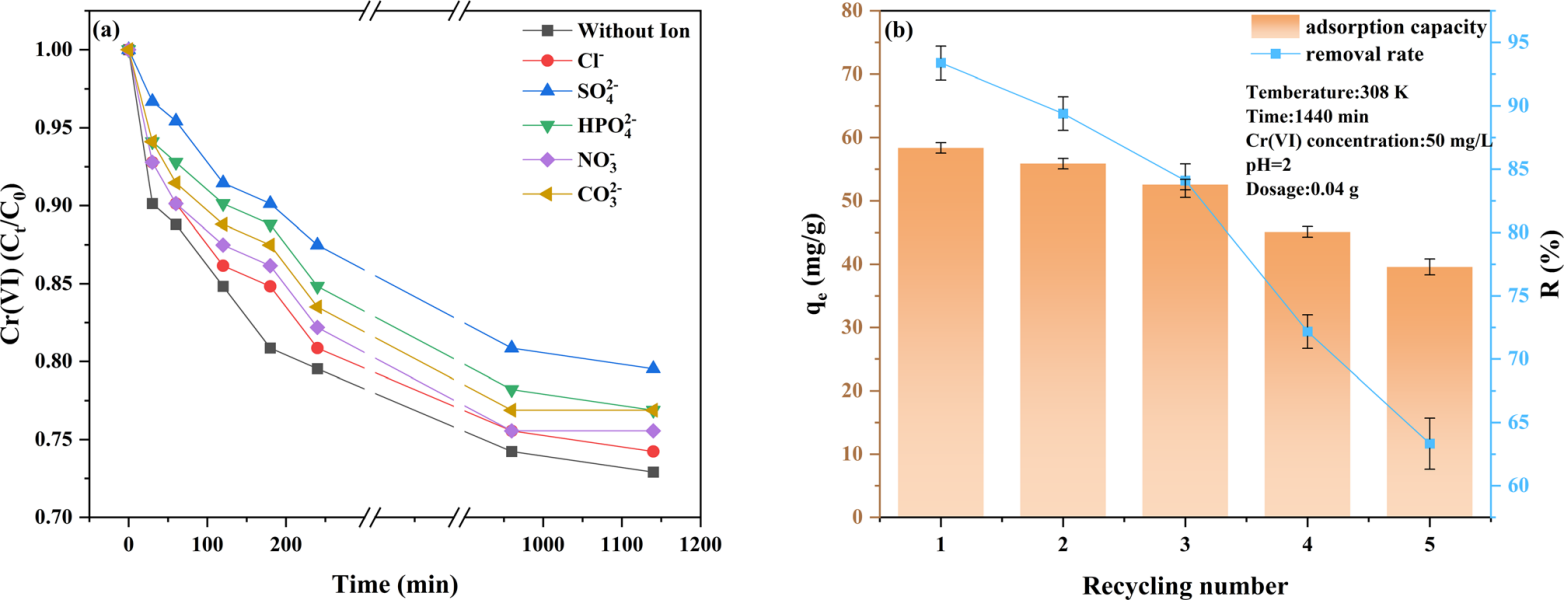
(a) Effect of different anions on Cr(vi) adsorption. Reaction conditions: Cr(vi) solution volume = 50 mL (50 mg L^−1^), adsorbent dosage = 0.02 g, anion concentration [(Cl^−^, SO^2−^_4_, NO^−^_3_, HPO^2−^_4_, CO^2−^_3_)] = 0.01 M; (b) recycling and regeneration performance.

The effects of five common anions (Cl^−^, SO_4_^2−^, NO_3_^−^, HPO_4_^2−^ and CO_3_^2−^) on the adsorption of Cr(vi) by FC were also studied. The results are shown in Fig. 8a. The presence of all anions inhibited the adsorption of Cr(vi) by FC, and their inhibitory effects were as follows: SO_4_^2−^ > HPO_4_^2−^ > CO_3_^2−^ > NO_3_^−^ > Cl^−^. Among them, SO_4_^2−^ has the most significant inhibitory effect on the adsorption of Cr(vi). This might be because SO_4_^2−^ can compete for adsorption on the FC surface through electrostatic interaction, thereby inhibiting the adsorption effect of Cr(vi).^40^ In addition, SO_4_^2−^ can form strong complexes with Cr(vi), resulting in a decrease in its affinity for the adsorption site.^41^ Further study of Table S3 reveals that in a solution containing CO_3_^2−^, carbonate ions combine with water molecules to form bicarbonate ions, resulting in an initial pH of 10.86, which is the highest value among all test conditions. Under such high pH conditions, the main form of Cr(vi) is CrO_4_^2−^, whose ion size is larger than that of HCrO_4_^−^. Compared with HCrO_4_^−^, the diffusion of CrO_4_^2−^ in solution is more difficult,^42^ which is not conducive to adsorption. The presence of CO_3_^2−^ inhibits the adsorption of Cr(vi) for two reasons: the dissociation of HCrO_4_^−^ into CrO_4_^2−^ generates electrostatic repulsion with the negatively charged FC surface.^43^ In addition, high concentrations of OH^−^ compete with Cr(vi) for adsorption sites on the FC surface. Although the presence of coexisting anions such as Cl^−^, SO_4_^2−^, HPO_4_^2−^, NO_3_^−^, and CO_3_^2−^ exerted varying degrees of competitive inhibition on Cr(vi) removal, the *C*_t_/C_0_ values in all systems were still maintained within the range of 0.74–0.80 after 1140 min of reaction, demonstrating that the material possessed good stability and strong resistance to interference under complex water conditions. The cyclic adsorption characteristics of FC on Cr(vi) are shown in Fig. 8b. After three cycles, the removal rate of Cr(vi) by FC decreased. This is because as the number of cycles increased, the effective active sites of FC gradually decreased. Despite this, after five cycles, the removal rate of Cr(vi) remained at 63.32%, indicating that FC has excellent cycling performance.

#### 3.2.3 Adsorption kinetics and adsorption isotherms

**Fig. 9:**
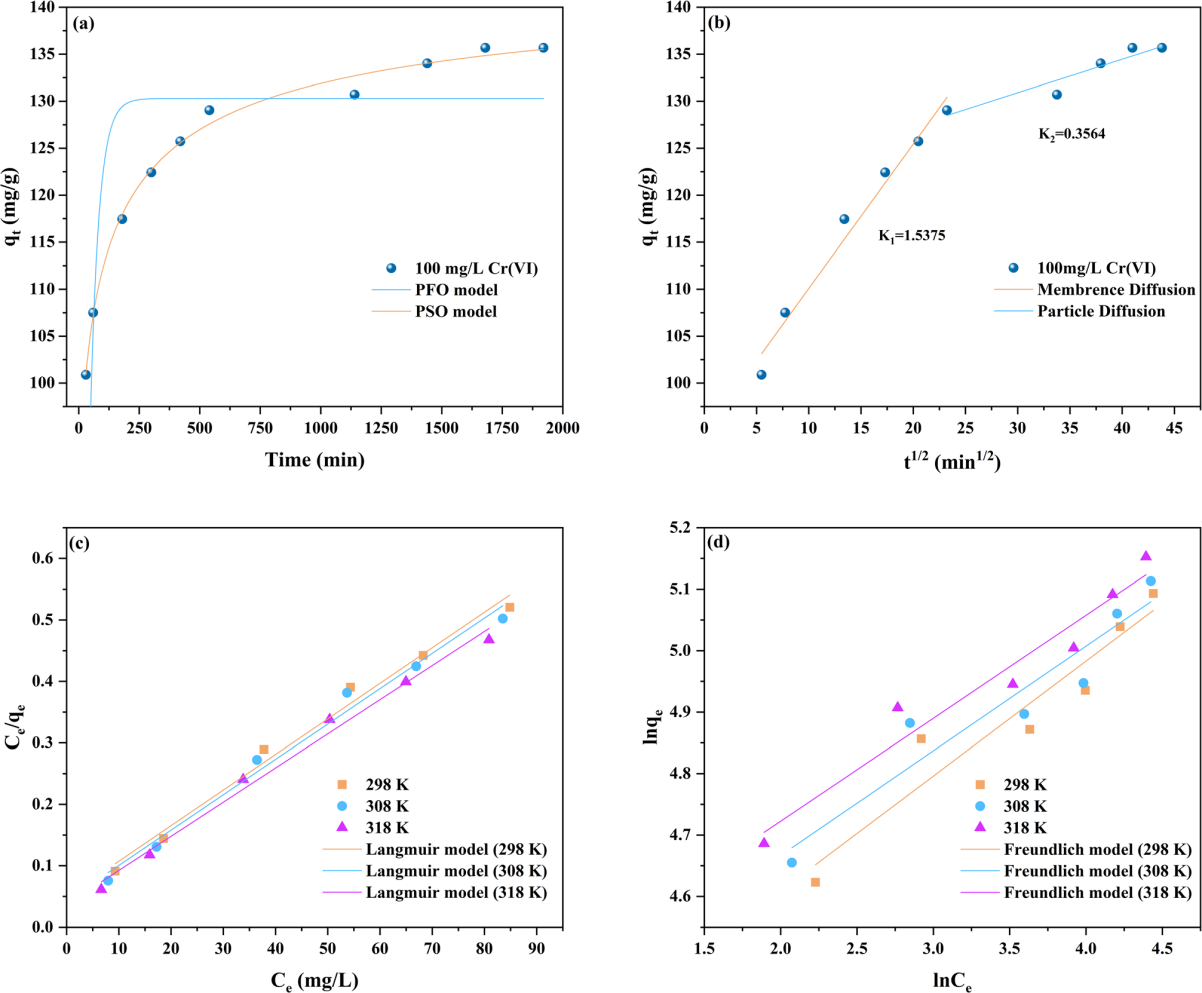
Adsorption kinetics models: (a) PFO and PSO models; (b) ID model; adsorption isotherms models: (c) Langmuir model; (d) Freundlich model.

To investigate the adsorption kinetics mechanism of Cr(vi) on FC, pseudo-first-order kinetics (PFO) and pseudo-second-order kinetics (PSO) were employed to analyse the adsorption experimental data. Concurrently, intra-particle diffusion (ID) was utilised to analyse the mass transfer kinetics process. The relevant equations are presented in Text S2, with the fitted parameters listed in Table S4. The correlation coefficient obtained from the PSO model in Fig. 9a (*R*^2^ = 0.9944) was higher than that from the PFO model (*R*^2^ = 0.4114), indicating that the adsorption process of Cr(vi) on FC aligns with the PSO model. Moreover, the theoretical equilibrium adsorption capacity calculated *via* the PSO model (*q*_e,cal_ = 136.3703 mg g^−1^) approximates the experimental value (*q*_exp_ = 135.6739 mg g^−1^) more closely, suggesting that chemisorption is the rate-limiting step of the process.^44^ Conversely, the intraparticle diffusion (ID) model in Fig. 9b reveals that the adsorption kinetics of Cr(vi) are jointly controlled by intraparticle diffusion and external surface diffusion, indicating that the overall mass transfer process involves multiple diffusion steps rather than a single rate-limiting stage.^45^ The first straight line corresponds to the external surface diffusion of Cr(vi) from the liquid phase to the FC surface, while the second line segment reflects the intraparticle diffusion behaviour of Cr(vi) into the FC's internal pores. The significantly steeper slope of the first line segment (*k*_id,1_ > *k*_id,2_) indicates that external surface diffusion proceeds at a faster rate.^46^ Consequently, the rate-limiting step during the initial adsorption phase is dominated by intra-particle diffusion. As the reaction progresses and the Cr(vi) concentration decreases, the concentration gradient between the adsorbate and adsorbent diminishes, weakening the external surface diffusion effect. Surface diffusion gradually becomes the rate-limiting factor. As Cr(vi) accumulates continuously within the pores, diffusion resistance increases, causing the adsorption rate to gradually decrease until dynamic equilibrium is reached. Furthermore, in the ID model, the boundary layer diffusion parameter *c*_2_ is greater than *c*_1_, indicating that boundary layer effects exert a more significant influence on the adsorption rate during intra-particle diffusion.^47^

To further investigate the adsorption mechanism, Langmuir and Freundlich isotherm models (Text S3) were employed to analyse the equilibrium adsorption data. The results are presented in Fig. 9c and d, with the fitted parameters listed in Table S5. Both models exhibited good fit, and the Freundlich unevenness coefficient (*n* > 1) indicated effective adsorption. The Langmuir model exhibited a high correlation coefficient (*R*^2^ > 0.9859), yielding a maximum adsorption capacity for Cr(vi) of 179.8561 mg g^−1^ and a peak *K*_L_ value of 0.1513. These results indicate that the adsorption process of Cr(vi) on FC can be described by a monolayer adsorption mechanism;^48^ interactions between adsorbate and adsorbent intensify at elevated temperatures, enhancing adsorption efficiency. Compared with other adsorbents, FC exhibits superior adsorption performance, as summarised in Table S6.

#### 3.2.4 Adsorption thermodynamics

To investigate the thermodynamic mechanisms of the adsorption process in depth, this study calculated and analysed the adsorption thermodynamic parameters, including the Gibbs free energy change (Δ*G*^*θ*^), enthalpy change (Δ*H*^*θ*^), and entropy change (Δ*S*^*θ*^). Detailed thermodynamic parameter data and analysis results are summarised in Table S7. The calculation formulas, data fitting methods, and relevant graphical representations are detailed in Text S4 and Fig. S2. The Δ*G*^*θ*^ values obtained at temperatures of 298 K, 308 K, and 318 K were all negative, indicating that the adsorption of Cr(vi) onto FC is a spontaneous process.^49^ Moreover, the absolute value of Δ*G*^*θ*^ increases with rising temperature, indicating that elevated temperatures favour the spontaneous adsorption reaction, thereby enhancing Cr(vi) adsorption capacity. The positive value of Δ*H*^*θ*^ (17.663 kJ mol^−1^) confirms the endothermic nature of the adsorption process, which is in good agreement with the results obtained from the isotherm studies. Furthermore, the magnitude of Δ*H*^*θ*^ suggests that the process is predominantly governed by physisorption.^50^ Furthermore, the positive Δ*S*^*θ*^ value corresponds to an increase in entropy during adsorption, indicating heightened disorder at the solid/liquid interface during Cr(vi) adsorption.^51^

### 3.3 Adsorption mechanism

To elucidate the physicochemical mechanisms governing Cr(vi) removal by FC, a series of characterisation analyses were conducted before and after adsorption. The N2 adsorption–desorption isotherms and pore structure analysis (Fig. S3) revealed that the specific surface area of FC decreased markedly from 630.308 m^2^ g^−1^ to 151.557 m^2^ g^−1^ following Cr(vi) adsorption, representing a reduction of approximately 76% (Table S8). More notably, the micropore-specific surface area diminished from 63.794 m^2^ g^−1^ to 0.00 m^2^ g^−1^, indicating that the microporous network was effectively saturated during the adsorption process. These observations provide compelling evidence that pore filling, particularly within the micropore domain, constitutes a principal physical adsorption mechanism.

**Fig. 10:**
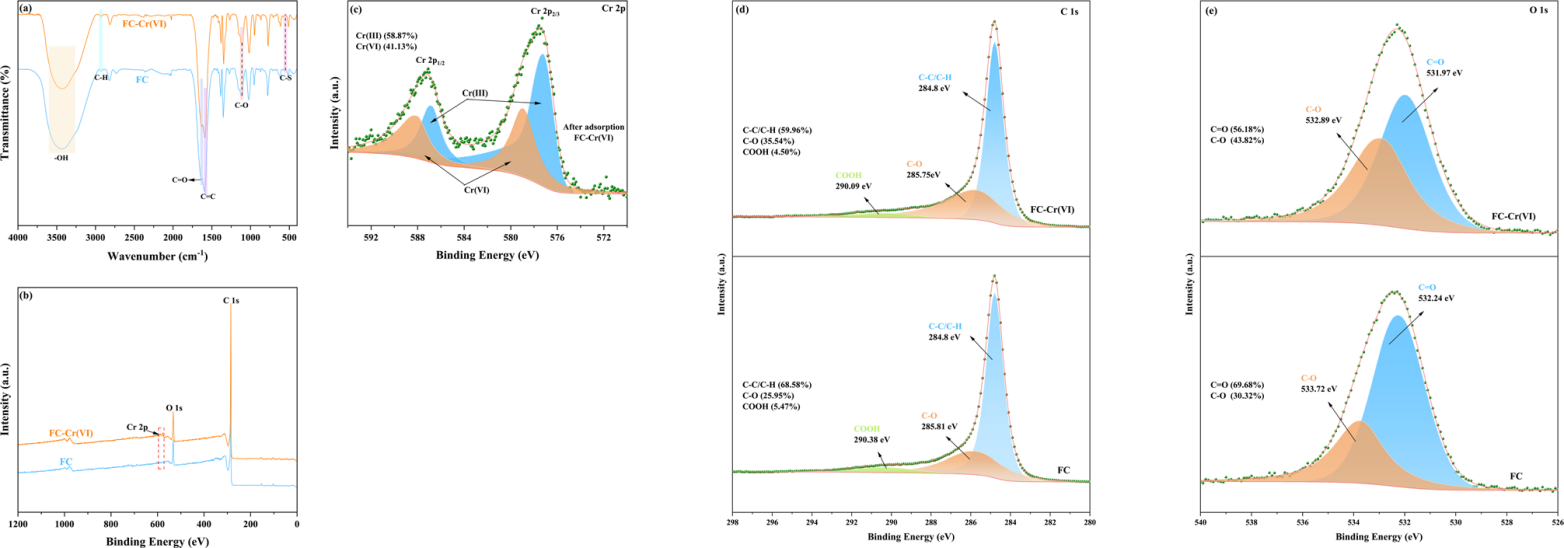
(a) FTIR spectra of FC and FC-Cr(vi); (b) XPS survey scan spectra of FC and FC-Cr(vi); (c) Cr 2p, (d) C 1s, and (e) O 1s high-resolution spectra.

Further mechanistic insight was obtained through comparative FTIR spectroscopy of FC before and after Cr(vi) adsorption (FC–Cr(vi)), as presented in Fig. 10a. The broad absorption band at 3440 cm^−1^, attributed to the O–H stretching vibration, confirms the presence of hydrophilic hydroxyl groups on the FC surface. These surface –OH groups facilitate hydrogen bonding with water molecules, thereby promoting interfacial wettability and expanding the accessible contact area in Cr(vi)-containing solution.^52^ Upon Cr(vi) adsorption, the intensity of this band decreased appreciably, suggesting that –OH groups are involved in electrostatic interactions with anionic Cr(vi) species (HCrO_4_^−^, Cr_2_O_7_^2−^, and CrO_4_^2−^) through electron donation and hydrogen bonding with the oxygen atoms of these anions.^53^ At pH = 2.0, which is below the point of zero charge (pH_pzc_) of FC, the adsorbent surface carries a net positive charge, thereby enhancing the electrostatic affinity towards Cr(vi) anions. In addition, the characteristic absorption band of the C–S bond shifted from 554 cm^−1^ to 542 cm^−1^ after adsorption, a red shift indicative of reduced vibrational force constants and diminished bond stability. This spectral displacement implies that the reductive C–S functionality is involved in the adsorption-coupled reduction of Cr(vi) to Cr(iii), as described by eqn (8):^54^

8 C–S + HCrO_4_^−^ + 3H^+^ → C–SO_2_ + Cr^3+^ + 2H_2_O

The collective shifts in peak positions of the principal functional groups (–OH, C–O, C=O, C–S, and C=C) confirm that both S- and O-containing surface functionalities play integral roles in the Cr(vi) adsorption process.

To quantify the surface chemical transformations, XPS was employed. The survey spectra (Fig. 10b) of FC prior to adsorption exhibited characteristic peaks for C 1s and O 1s. After Cr(vi) adsorption, the emergence of a distinct Cr 2p signal confirmed the successful immobilisation of chromium species on the FC surface. High-resolution fitting of the Cr 2p spectrum (Fig. 10c) resolved four constituent peaks: Cr(iii) 2p_3/2_ (577.21 eV), Cr(iii) 2p_1/2_ (586.81 eV), Cr(vi) 2p_3/2_ (578.96 eV), and Cr(vi) 2p_1/2_ (588.06 eV).^55^ Quantitative deconvolution revealed that the adsorbed chromium comprised 58.87% Cr(iii) and 41.13% Cr(vi), demonstrating that a substantial fraction of Cr(vi) underwent reductive conversion to Cr(iii) during the adsorption process. The Cr(iii) species generated *in situ* are subsequently retained on the FC surface through surface complexation with oxygen-containing functional groups (C–O and C=O), which accounts for the strong Cr(iii) signal detected by XPS.^56^

The C 1s XPS spectrum (Fig. 10d) was deconvoluted into three components: C–C/C–H (284.80 eV), C–O (285.81 eV), and COOH (290.38 eV).^57^ Following Cr(vi) adsorption, the relative proportions of C–C/C–H and COOH decreased from 68.58% and 5.47% to 59.96% and 4.50%, respectively, while the C–O fraction increased correspondingly from 25.95% to 35.54%. These changes are consistent with the participation of aromatic C–C/C–H and carboxyl (COOH) functionalities in electron–transfer reactions with Cr(vi), both of which possess relatively low excitation energy barriers and exhibit electron-donating capacity.^58^ The net increase in C–O content reflects the oxidation of carbon-bound hydrogen and carbon–carbon bonds during the reductive conversion of Cr(vi). The O 1s XPS spectrum (Fig. 10e) was fitted with two components assigned to C–O (532.89 eV) and C=O (531.97 eV).^59^ After adsorption, the proportion of C=O decreased, whereas C–O increased from 30.32% to 43.82%. This trend is consistent with the consumption of C=O groups *via* their involvement in Cr(vi) reduction. At pH = 2.0, Cr(vi) and Cr(iii) predominantly exist as HCrO_4_^−^ and Cr^3+^, respectively, and the following surface redox reactions have been proposed:^60^

9 COOH + 4HCrO_4_^−^ + 16H^+^ → C–H + 4Cr^3+^ + 10H_2_O + 4O_2_

10 C–H + 2HCrO_4_^−^ + 8H^+^ → C–OH + 2Cr^3+^ + 5H_2_O + O_2_

11 C–C + 2HCrO_4_^−^ + 8H^+^ → 2C–OH + 2Cr^3+^ + 4H_2_O + O_2_

12 3C–OH + HCrO_4_^−^ + 4H^+^ → 3C=O + Cr^3+^ + 4H_2_O

**Fig. 11:**
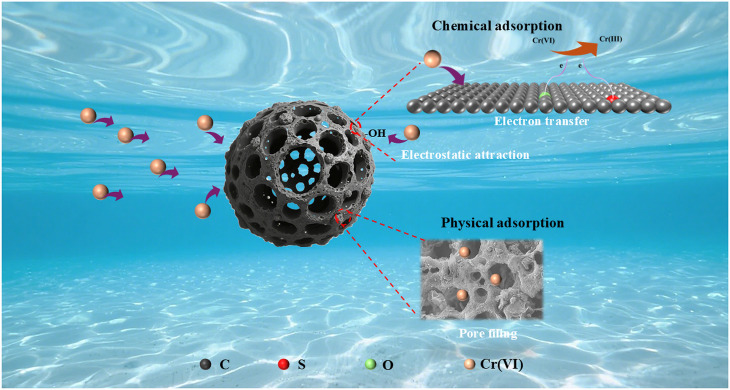
Adsorption mechanism for Cr(vi) on FC.

Integrated analysis of adsorption kinetics, isotherm modelling, and spectroscopic characterisation indicates that the removal of Cr(vi) by FC is governed by a synergistic interplay of physical adsorption and chemical interactions (Fig. 11). The well-developed microporous structure and high specific surface area of FC provide abundant adsorption sites, enabling Cr(vi) anions to rapidly diffuse into the pore network and accumulate on the surface through a pore-filling mechanism. When the solution pH is lower than the pHpzc of FC, the positively charged surface generates strong electrostatic attraction toward the predominant Cr(vi) anions, including HCrO_4_^−^, Cr_2_O_7_^2−^, and CrO_4_^2−^. Meanwhile, surface –OH groups can form hydrogen bonds with these oxygen-containing anions, further strengthening the interfacial interactions and promoting their adsorption. As the adsorption process proceeds, surface chemical reactions become increasingly significant. Electron-rich functional groups on the FC surface (such as C–S, C–C/C–H, COOH, and C=O) act as electron donors in redox reactions, facilitating the partial reduction of Cr(vi) to the less toxic Cr(iii). In addition, Cr(vi) anions and the *in situ* generated Cr(iii) species can form stable inner-sphere surface complexes with oxygen-containing functional groups on the FC surface (–OH, C–O, and C=O), resulting in the effective immobilisation of chromium species. Overall, pore filling, electrostatic attraction coupled with hydrogen bonding, surface redox reactions, and surface complexation collectively constitute the synergistic mechanism responsible for Cr(vi) removal by FC.

## 4 Conclusion

In this study, a sequential acid–alkali activation strategy was proposed to convert coal gasification fine slag into a high-performance hierarchically porous carbon (FC) for efficient Cr(vi) removal. During the activation process, mineral ash was removed by acid treatment to release encapsulated carbon domains, while subsequent alkali activation disrupted silicate frameworks and reconstructed interconnected micro–meso–macroporous networks, resulting in a significantly increased specific surface area and abundant surface active sites. Consequently, the obtained FC possessed a well-developed microporous structure and rich oxygen- and sulfur-containing functional groups, providing favorable conditions for Cr(vi) adsorption and transformation. Adsorption kinetics and isotherm analyses indicated that Cr(vi) uptake followed monolayer adsorption behavior and that chemisorption served as the rate-limiting step, while thermodynamic results suggested that the adsorption process was spontaneous and endothermic. The presence of coexisting anions exerted varying degrees of competitive inhibition on Cr(vi) adsorption, mainly due to competitive adsorption, complexation effects, and pH-induced changes in Cr(vi) speciation. Good structural stability was preserved after repeated regeneration cycles. Integrated analyses of adsorption kinetics, isotherm modeling, and spectroscopic characterization further revealed that Cr(vi) removal was governed by multiple synergistic mechanisms, including pore-filling-induced enrichment, electrostatic attraction and hydrogen bonding under acidic conditions, as well as surface redox reactions and complexation. Overall, the structure–function relationship governing Cr(vi) removal by coal gasification fine slag-derived porous carbon was elucidated, providing a sustainable and scalable pathway for upgrading industrial solid waste into high-value functional materials for heavy-metal remediation.

## Author contributions

Lijuan Bai: conceptualization, methodology, investigation, writing – original draft; Hua Wang: funding acquisition, supervision, project administration, resources, writing – review & editing; Kaipeng Guo: data curation, software, formal analysis; Xia Li: validation, investigation; Kaiwen Bai: investigation, visualization; Zhengyan Shi: methodology, resources; Rui Dang: validation, funding acquisition.

## Conflicts of interest

The authors declare that they have no known competing financial interests or personal relationships that could have appeared to influence the work reported in this paper.

## Supplementary Material

RA-016-D6RA02711C-s001

## Data Availability

The data supporting this article have been included as part of the supplementary information (SI). Supplementary information is available. See DOI: https://doi.org/10.1039/d6ra02711c.
