# Supplementary material for: Acid-base synergistic activation of coal gasification fine slag into hierarchical porous carbon for enhanced Cr(vi) adsorption and reduction
Source: RSC Adv. 2026 May 18;16(29):26473–87. doi: 10.1039/d6ra02711c (PMC13185810; doi:10.1039/d6ra02711c)
Supplement: RA-016-D6RA02711C-s001 [file RA-016-D6RA02711C-s001.pdf]

Supplementary Information (SI) for RSC Advances.
This journal is © The Royal Society of Chemistry 2026

Acid-Base Synergistic Activation of Coal Gasification Fine Slag into Hierarchical Porous Carbon for Enhanced Cr(VI) Adsorption and Reduction

Lijuan Bai[1], Hua Wang[1,2], Kaipeng Guo[1], Xia Li[1], , Kaiwen Bai[1], Zhengyan Shi[1], Rui Dang[1]*
[1] College of Chemistry and Chemical Engineering, Yulin University, Yulin City 719000, China
[2] Shaanxi Provincial Key Laboratory of Clean Utilization of Low-Modified Coal, Yulin University, Yulin City 719000, China
[*]Corresponding author at:College of Chemistry and Chemical Engineering, Yulin University,Chongwen Road No.51, Yulin City 719000, Shaanxi Province, China. E-mail address: 99452715@qq.com (H. Wang)

Text S1: FHH model

Based on low-temperature nitrogen adsorption data, the fractal dimension (D) of four samples was calculated using the Frenkel-Halsey-Hill (FHH) fractal model. This parameter reflects the degree of pore formation within the material. D was calculated using Equations (1) and (2) [1]:

$$\ln(V) = b + A\ln\left(\ln\frac{P_0}{P}\right) \quad (1)$$

$$D = A + 3 \quad (2)$$

Where V denotes the adsorption volume of N_2 at equilibrium pressure P, and P_0 represents the saturation pressure of N_2. A is the power-law exponent associated with the fractal dimension (D), and b is a constant. The value of D ranges from 2 to 3.

Text S2: Adsorption kinetic models [2]

PFO: $$q_t = q_e(1 - e^{-k_1 t}) \quad (3)$$

PSO: $$q_t = \frac{q_e^2 k_2 t}{1 + q_e k_2 t} \quad (4)$$

ID: $$q_t = k_{id} t^{1/2} + c \quad (5)$$

q_e (mg/g) denotes the equilibrium adsorption capacity; k_1 (1/min) and k_2 (g/(mg·min)) represent the adsorption rate constants for the PFO and PSO models respectively; kid (mg/(g·min$^{1/2}$)) indicates the particle internal diffusion coefficient; c (mg/g) reflects the influence of the boundary layer on the adsorption rate, with higher c values signifying greater impact.

Text S3: Adsorption isotherm models [3]

Langmuir: $$\frac{C_e}{q_e} = \frac{C_e}{q_m} + \frac{1}{K_L q_m} \qquad (6)$$

Freundlich: $$\ln q_e = \ln K_F + \frac{1}{n} \ln C_e \qquad (7)$$

Here, q_m (mg/g) denotes the maximum adsorption capacity. The constant K_L (L/mg) represents the Langmuir equilibrium constant. The parameter K_F ($mg/g(L/mg)^{1/n}$) is the Freundlich constant; a higher K_F value indicates a stronger adsorption reaction. The variable n is the Freundlich heterogeneity factor; when $n > 1$, it signifies high adsorption efficiency, whereas lower values indicate poor adsorption performance.

Text S4: Adsorption thermodynamics [4]

$$K_d = \frac{q_e}{C_e} \tag{8}$$

$$\ln K_d = \frac{\Delta S^\theta}{R} - \frac{\Delta H^\theta}{RT} \tag{9}$$

$$\Delta G^\theta = -RT \ln K_d \tag{10}$$

Here, K_d denotes the thermodynamic equilibrium constant; $\triangle H^\theta$ and $\triangle S^\theta$ are derived from the slope and intercept of $\ln K_d$ versus T^{-1}. R (8.314 J/mol·K) represents the molar gas constant.

Table S1 Fractal dimensions of FHH model for CGFS, FC and CGFS-H.

samples	P/P_0=0~0.4			P/P_0=0.4~1		
	A_1	D_1	R_1^2	A_2	D_2	R_2^2
CGFS	-0.1887	2.8113	0.9652	-0.4705	2.5295	0.9992
CGFS-H	-0.1723	2.8277	0.9415	-0.4456	2.5544	0.9995
FC	-0.1332	2.8668	0.9796	-0.3190	2.6810	0.9904

Table S2 Carbon microstructure parameters of CGFS and FC.

samples	I_D/I_G	A_{D1}/A_{all}	A_G/A_{all}	$A_{(D3+D4)}/A_{all}$
CGFS	0.6997	0.2463	0.3520	0.4016
FC	0.9827	0.3223	0.3279	0.3497

Table S3 pH value changes of various anions before and after adsorption.

pH	Cl^-	SO_4^{2-}	NO_3^-	HPO_4^{2-}	CO_3^{2-}	Without Ion
Initial	5.19	4.85	4.91	4.43	10.86	5.28
Final	5.76	5.56	5.57	4.63	10.66	5.92

Table S4 Adsorption kinetic parameters fitted based on PFO, PSO and ID models.

models	Parameters	C_0,Cr(VI)
		100 mg/L
	$q_{e,exp}$ (mg/g)	135.6739
	$q_{e,cal}$ (mg/g)	130.1276
PFO	k_1 (1/min)	0.0278
	R^2	0.4114
	$q_{e,cal}$ (mg/g)	136.3703
PSO	k_2 (g/mg/min)	0.0073
	R^2	0.9944
	$k_{id,1}$ (mg/(g · $min^{1/2}$)	1.5375
	c_1	94.7002
ID	R^2	0.9772
	$k_{id,2}$ (mg/(g · $min^{1/2}$)	0.3564
	c_2	120.2107
	R^2	0.9029

Table S5 Adsorption isotherm parameter of Cr(VI) by FC.

models	Parameters	Temperature		
		298 K	308 K	318 K
Langmuir	q_m (mg/g)	172.7115	173.9130	179.8561
	K_L	0.1170	0.1339	0.1513
	R^2	0.9871	0.9859	0.9902
Freundlich	$K_{F(}mg/g(L/mg)^{1/n})$	69.0361	75.6290	80.4310
	n	5.3444	5.8685	5.9684
	R^2	0.9164	0.9065	0.9483

Table S6 Comparison of the adsorption capacity of FC and other adsorbents for Cr(VI).

Adsorbent	Temperature (K)	q_{max} (mg/g)	Reference
NiCo-LDHM	298 K	132.00	[5]
PACFs	298 K	93.20	[6]
Al/BC	298 K	176.23	[7]
Fe_3O_4@SiO_2-UiO-66-EDTA	298 K	75.11	[8]
FC	298 K	172.71	This study

Table S7 Adsorption thermodynamic parameter.

T (K)	ΔG^{θ} (KJ/mol)	ΔH^{θ}(KJ/mol)	ΔS^{θ}((J/(mol·K))
298	-7.674		
308	-8.476	17.663	84.974
318	-9.376		

Table S8 Specific surface area and pore distribution of FC and FC-Cr(VI)

samples	Specific surface area (m^2/g)	Micropore surface area (m^2/g)	Mesopore Surface area (m^2/g)	Total pore volume (cm^3/g)
FC	630.308	63.794	250.078	0.728
FC-Cr(VI)	151.577	0.000	73.918	0.205

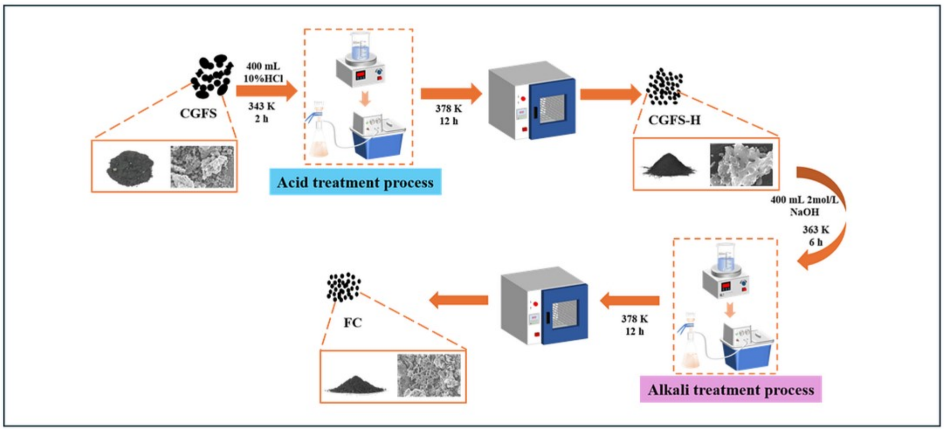

Figure S1. Schematic diagrams of the preparation processes for FC and CGFS-H

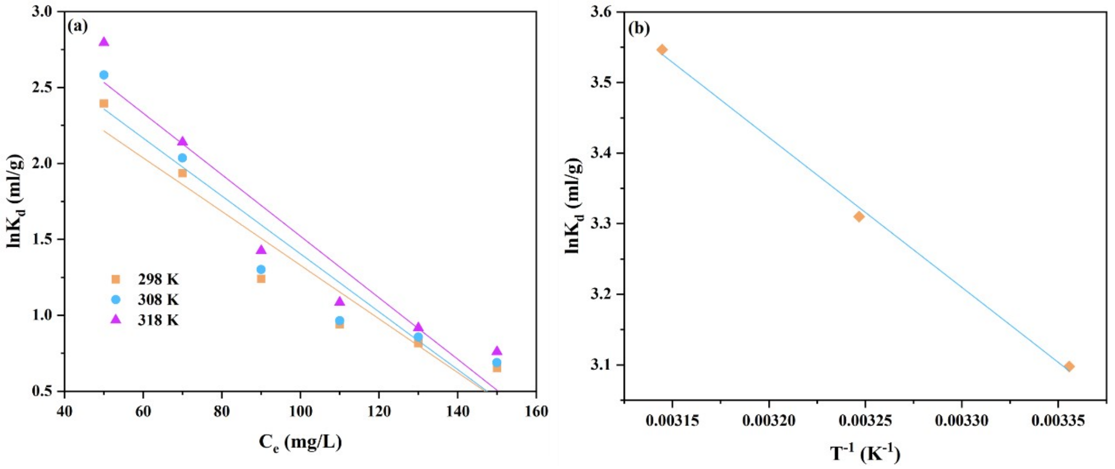

Figure S2 (a)The relationship curve between $lnK_d$and C_e; (b)The relationship curve between lnK_d and T^{-1}

Figure S3 (a) N_2 adsorption-desorption isotherms of FC and FC-Cr(VI); (b) pore size distribution

References

[1] X. Li, Q. Wu, D. Chen, R.S. Tamjidur, P. Fan, G. Wang, W. Zhang, Efficient chromium Cr (VI) removal from wastewater through modified cycad's leaf biochar: insights into adsorption-reduction mechanisms and kinetic analysis, Biomass and Bioenergy 200 (2025) 107994.

[2] F. Liu, Y. Luo, B. Hu, Simultaneous elimination of U(VI) and Eu(III) by phytic acid decorated MXenes@MOFs composites in water: Performance, kinetics and mechanism, Separation and Purification Technology 327 (2023) 124912.

[3] H. Ji, H. Wang, Q. Yang, X. Xie, D. Wang, B. Wu, Simultaneous removal of Cr(VI), Cd(II) and As(III) in groundwater by ZVI-biochar based composite: Synergy, performance and mechanism, Process Safety and Environmental Protection 201 (2025) 107460.

[4] L. Mingzhe, Y. Zhang, L. Xiaoyan, Performance and mechanism of uranium (VI) removal from aqueous solution by zero-valent nickel@hollow glass particles (ZVNi@HGP), Physica Scripta 99 (2024) 075961.

[5] M. Li, X. Chen, J. He, S. Liu, Y. Tang, X. Wen, Porous NiCo-LDH microspheres obtained by freeze-drying for efficient dye and Cr(VI) adsorption, Journal of Alloys and Compounds 976 (2024) 173107.

[6] S.B. Kim, M. Kim, J. Lee, H. Choi, S.Y. Lee, S.J. Park, Impact of pore structure in pitch-based activated carbon fibers on Cr(VI) adsorption behaviors, Applied Surface Science Advances 29 (2025) 100835.

[7] Z. Yang, H. Wu, X. Yan, D. Bekchanov, D. Kong, X. Su, Preparation of Al-doped carbon materials derived from artificial potassium humate prepared from waste cotton cloth and their excellent Cr(VI) adsorption performance, Colloids and Surfaces A: Physicochemical and Engineering Aspects 699 (2024) 134721.

[8] B. Hou, X. Yang, L. Wang, T. Shi, M. Akram, L. Wang, J. Wan, B. Gao, J. Pan, Fe doping enhanced Cr(VI) adsorption efficiency of cerium-based adsorbents: Adsorption behaviors and inner removal mechanisms, Journal of Colloid and Interface Science 67 (2024).
